# Toll-like receptor 2 (*TLR2*) gene polymorphisms are not associated with sarcoidosis in the Japanese population

**Published:** 2011-03-15

**Authors:** Mayuki Sato, Tatsukata Kawagoe, Akira Meguro, Masao Ota, Yoshihiko Katsuyama, Mami Ishihara, Kenichi Namba, Nobuyoshi Kitaichi, Shin-ichiro Morimoto, Toshikatsu Kaburaki, Yasutaka Ando, Shinobu Takenaka, Shigeaki Ohno, Hidetoshi Inoko, Nobuhisa Mizuki

**Affiliations:** 1 Department of Ophthalmology and Visual Science, Yokohama City University Graduate School of Medicine, Yokohama, Kanagawa, Japan; 2 Department of Legal Medicine, Shinshu University School of Medicine, Matsumoto, Nagano, Japan; 3 Department of Pharmacy, Shinshu University Hospital, Matsumoto, Nagano, Japan; 4 Hiyoshi Eye Clinic, Yokohama, Kanagawa, Japan; 5 Department of Ophthalmology, Hokkaido University Graduate School of Medicine, Sapporo, Hokkaido, Japan; 6 Department of Ophthalmology, Health Sciences University of Hokkaido, Sapporo, Hokkaido, Japan; 7 Department of Ocular Inflammation and Immunology, Hokkaido University Graduate School of Medicine, Sapporo, Hokkaido, Japan; 8 Division of Cardiology, Department of Internal Medicine, Fujita Health University School of Medicine, Toyoake, Aichi, Japan; 9 Department of Ophthalmology, University of Tokyo School of Medicine, Tokyo, Japan; 10 Department of Ophthalmology, Kitasato Institute Hospital, Tokyo, Japan; 11 Department of Ophthalmology, Keio University School of Medicine, Tokyo, Japan; 12 Department of Respiratory Diseases, Kumamoto City Hospital, Kumamoto, Japan; 13 Department of Basic Science and Molecular Medicine, Tokai University School of Medicine, Isehara, Kanagawa, Japan

## Abstract

**Purpose:**

Sarcoidosis is a systemic inflammatory disease characterized by the formation of non-caseating granulomas, with varied clinical manifestations. The common etiology of sarcoidosis is uncertain, but it is thought to be triggered by an exogenous antigenic stimulus, such as some bacterial proteins. Toll-like receptors (TLRs) recognize microbial components and elicit innate as well as adaptive immune responses. It has been reported that polymorphisms in *TLR2* might be important in a small group of Caucasian sarcoidosis patients. The present study aimed to establish whether these findings are relevant to the Japanese population.

**Methods:**

We genotyped 5 single-nucleotide polymorphisms (SNPs) in *TLR2* and assessed the allelic diversity between 257 Japanese sarcoidosis patients and 193 Japanese healthy controls.

**Results:**

No significant differences in the frequency of *TLR2* alleles and haplotypes in the sarcoidosis cases were found in comparison with the controls. However, marginal associations were observed for *TLR2* at rs3804099 and rs3804100 in sarcoidosis patients with cutaneous manifestations.

**Conclusions:**

Our results suggest that *TLR2* polymorphisms are not significantly related to the pathogenesis of sarcoidosis in the Japanese population.

## Introduction

Sarcoidosis is a systemic inflammatory disorder resulting in non-caseating granulomas in multiple organs, such as: lung, skin, eye, lymph nodes, central and peripheral nervous system, and heart [1-3]. Japanese patients have a higher likelihood of ocular involvement compared with other ethnic groups [4]. Ocular manifestation is one of the most common presentation in Japanese sarcoidosis patients [5]. Granulomatous inflammation can occur in any layer of the eyeball, and leads to wide variety of ocular pathology, including uveitis. A survey of almost 3,000 Japanese patients diagnosed with uveitis found that sarcoidosis was the most frequent (13.3%) cause of non-idiopathic uveitis [6].

The exact cause of sarcoidosis is unknown, but the fact that the frequency and course of the disease varies widely among racial groups suggests that genetic factors may be the basis of disease susceptibility. African Americans are more commonly and severely affected by sarcoidosis than Caucasian Americans. The annual sarcoidosis incidence for African Americans is threefold higher, compared with Caucasian Americans; at 35.5 versus 10.9 cases per 100,000, respectively [7]. In the Swedish, another ethnic group, the annual incidence of sarcoidosis is also high [8]. In Japan, the annual estimated prevalence is 1.01 per 100,000 [5]. In Korea, the reported incidence rate is similarly low [9].

Environmental factors are also thought to contribute to the disease progression. The DNA of *Mycobacterium tuberculosis* and *Propionibacterium acnes* has been detected in some sarcoid lesions by using polymerase-chain-reaction (PCR) methods [10-13]. Recent studies have also shown that the serum of some sarcoidosis patients contains antibodies against mycobacterial antigens [14]. These studies suggest that bacterial infections can affect the development of sarcoidosis.

Toll-like receptors (TLRs) recognize microbial components and elicit innate as well as adaptive immune responses. Stimulation with TLR ligands induces the production of proinflammatory cytokines and type I interferons in cells of the innate immune system through intracellular signaling cascades [15-17]. Accumulating data suggest that *TLR* polymorphisms are closely associated with many autoimmune diseases [18-20]. Among the TLR family members, TLR2 recognizes multiple components of several bacterial cell walls, including peptidoglycans and lipoproteins from the cell wall of several bacteria and mycoplasma, by forming a heterodimer with either TLR1 or TLR6, and plays a critical role in the activation of innate immunity [21,22]. Polymorphisms in *TLR2* are associated with impaired responses to bacterial infection in human [23-27]. Recently, Veltkamp et al. [28] reported that they found the single nucleotide polymorphisms (SNPs) located in the *TLR2* promoter lesion (rs4696480) was associated with sarcoidosis in a Dutch Caucasian population, but could not confirm this in their validation cohort. They inferred from these findings that a *TLR2* variant could play a role in a small percentage of patients. The association between *TLR2* polymorphisms and sarcoidosis needs to be confirmed by further replication studies, particularly in other ethnic groups. In the present study, we therefore evaluated the association of multiple SNPs in *TLR2* in Japanese patients.

## Methods

### Subjects

Two hundred fifty-seven unrelated patients with a diagnosis of sarcoidosis and 193 healthy controls were recruited from Yokohama City University, Hokkaido University, Fujita Health University, Tokyo University, Keio University, and Kumamoto City hospital. All patients and control participants were of Japanese ethnicity. Sarcoidosis patients were diagnosed according to the diagnostic criteria developed by the Japanese Society of Sarcoidosis and Other Granulomatous Disorders (JSSOG) previously described [29]. Uveitis with sarcoidosis was assessed based on the “Guidelines for Diagnosis of Ocular Lesions in Sarcoidosis” prepared by the JSSOG. The ocular features of sarcoidosis were defined as granulomatous uveitis plus two or more of the following: infiltration of the anterior chamber (mutton-fat keratic precipitates/iris nodules), trabecular meshwork nodules and/or tent-shaped peripheral anterior synechia, masses of vitreous opacities (snowball-like or string of pearls-like appearance), periphlebitis with perivascular nodules; multiple candle-wax type chorioretinal exudates and nodules, and/or laser photocoagulation spot-like chorioretinal atrophy. All subjects had a similar social background and resided in the same urban area. The research methods were in compliance with the guidelines of the Declaration of Helsinki. Details of the study were explained to all patients and controls, and valid consent for genetic screening was obtained.

### Analysis of *TLR2* polymorphisms

**Figure 1:**
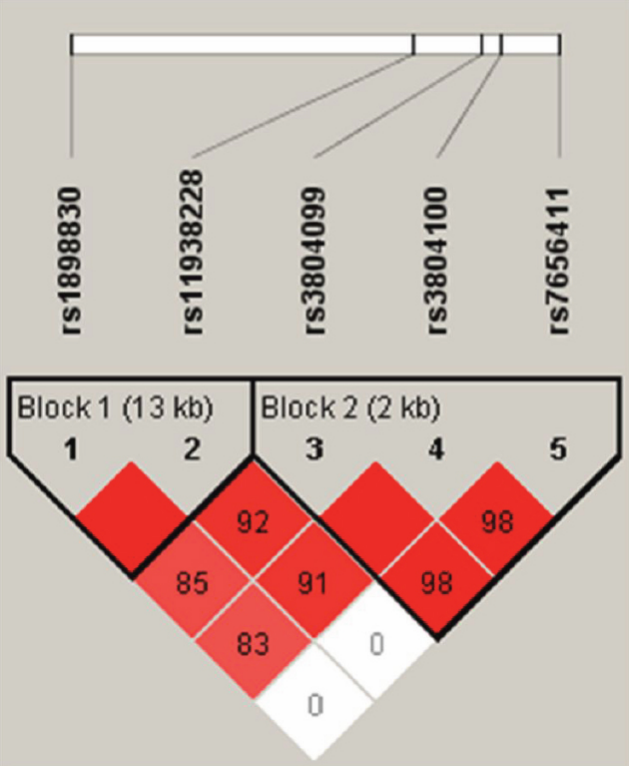
Linkage disequilibrium plot of five SNPs of *TLR2* in sarcoidosis patients and healthy controls. The schematic of the *TLR2* gene is shown as a black line, with boxes representing its three exons. The locations of the selected SNPs are indicated by the dotted lines. The Haplotype blocks were determined using the Haploview 4.2 software. Each box provides estimated statistics of the coefficient of determination, with brighter red representing a stronger Linkage disequilibrium. Values in squares represent pairwise D’ values.

Peripheral blood lymphocytes were collected, and genomic DNA was extracted from peripheral blood cells using the QIAamp DNA Blood Maxi Kit (Qiagen, Tokyo, Japan). We evaluated five single-nucleotide polymorphisms (SNPs): rs1898830, rs11938228, rs3804099, re3804100, and rs7656411 (Figure 1 and Table 1). These SNPs had minor allele frequencies (>5%) from the National Center for Biotechnology Information db SNP. Genotyping of all SNPs was performed using the TaqMan 5′ exonuclease assay using primers supplied by Applied Biosystems (Foster City, CA). Probe fluorescence signals were detected by TaqMan Assay for real-time PCR (7500 Real Time PCR System; Applied Biosystems) following the manufacturer’s instructions.

**Table 1:** Allele frequencies of SNPs of *TLR2* among sarcoidosis patients and controls.

** **	** **	** **	** **	**Minor allele frequency, n (%)**		** **	** **
**dbSNP**	**Alleles (1/2)**	**Position (bp)**	**Gene location**	**Cases (n=257)**	**Controls (n=193)**	**OR**	**p value**
rs1898830	A/G	154,827,903	Intron	232 (45.1)	185 (47.9)	0.89	0.41
rs11938228	C/A	154,841,396	Intron	228 (44.4)	180 (46.6)	0.91	0.50
rs3804099	T/C	154,844,106	Exon	165 (32.1)	108 (28.0)	1.22	0.18
rs3804100	T/C	154,844,859	Exon	155 (30.2)	97 (25.1)	1.29	0.097
rs7656411	G/T	154,847,105	3′UTR	218 (42.4)	168 (43.5)	0.96	0.74

In the “Alleles” column, 1 indicates the major allele and 2 indicates the minor allele. The position reflects the distance from short-arm telomere. p-values were caluculated by χ^2^ test 2×2 contingency table. bp, base pairs. OR, odds ratio.

### Statistical analysis

Hardy–Weinberg equilibrium was tested for each SNP among the controls. Differences in allele and genotype frequencies between cases and controls were assessed by the χ^2^ test. The Haploview 3.32 (Daly Lab at the Broad Institute, Cambridge, MA) program was used to compute pair-wise linkage disequilibrium (LD) statistics [30]. Standardized disequilibrium (D’) value was plotted, and LD blocks were defined according to the criteria [31]. Haplotype frequencies were estimated using an accelerated expectation-maximization algorithm similar to the partition-ligation-expectation- maximization method [32]. P values <0.05 were considered statistically significant. The Bonferroni method was used to correct multiple comparisons.

## Results

We genotyped five common SNPs in the *TLR2* gene: rs1898830, rs11938228, rs3804099, re3804100, and rs7656411 (Figure 1 and Table 1). All five SNPs were in Hardy–Weinberg equilibrium in the controls (data not shown). The minor allele frequencies of all SNPs were over 5% in the control group (Table 1). In this study, we did not examine the polymorphism of rs4696480, because there were no data for this minor allele in the Japanese HapMap database.

Linkage disequilibrium (LD) blocks of five SNPs in *TLR2* were defined (Figure 1). The *TLR2* region was divided into two haplotype blocks, with substantial LD among the SNPs of both blocks (block 1: D’≧1.00; block 2: D’≧0.98). The allele frequencies of the five SNPs in both the cases and controls are listed in Table 1, and genotype frequencies are listed in Table 2. No statistically significant association was observed for any of the SNPs between the cases and controls (p>0.05). We analyzed clinical features according to five SNPs. In a stratified analysis according to lesion location, which included the eye, lungs, heart, and nerves, none of these clinical features were found to be significantly associated with five SNPs (Table 3). Meanwhile, in 51 patients who had dermatitis, the minor allele frequencies of rs3804099 and rs3804100 were higher when compared with the frequency in 193 controls (p=0.021; p=0.013). However, these statistical differences disappeared after the Bonferroni correction was applied (p>0.05).

**Table 2:** Genotype frequencies of five SNPs of the *TLR2* gene in sarcoidosis patients and controls.

**SNP**	**Genotype**	**Cases, n (%)**	**Controls, n (%)**	**p value**
rs1898830	AA	77 (30.0)	53 (27.5)	0.69
	AG	128 (49.8)	95 (49.2)	
	GG	52 (20.2)	45 (23.3)	
rs11938228	CC	79 (30.7)	56 (29.0)	0.76
	CA	128 (49.8)	94 (48.7)	
	AA	50 (19.5)	43 (22.3)	
rs3804099	TT	116 (45.1)	99 (51.3)	0.39
	TC	117 (45.5)	80 (41.5)	
	CC	24 (9.3)	14 (7.3)	
rs3804100	TT	123 (47.9)	107 (55.4)	0.24
	TC	113 (44.0)	75 (38.9)	
	CC	21 (8.2)	11 (5.7)	
rs7656411	GG	86 (33.5)	62 (32.1)	0.95
	GT	124 (48.2)	94 (48.7)	
	TT	47 (18.3)	37 (19.2)	

p values were calculated using the χ^2^ test 3×2 contingency table.

**Table 3:** *TLR2* SNPs allele frequencies among sarcoidosis patients inflammatory sites and controls.

** **	** **	**Minor allele frequency, n(%)**						
** **	** **	** **	**Patients**					
**SNP**	**Alleles (1/2)**	**Controls (n=193)**	**Cases (n=257)**	**Eye (n=211)**	**Lungs (n=138)**	**Skin (n=51)**	**Heart (n=49)**	**Nerve (n=12)**
rs1898830	A/G	185 (47.9)	232 (45.1)	189 (44.8)	131 (47.5)	40 (39.2)	44 (44.9)	11 (45.8)
rs11938228	C/A	180 (46.6)	228 (44.4)	186 (44.1)	128 (46.4)	38 (37.3)	43 (43.9)	11 (45.8)
rs3804099	T/C	108 (28.0)	165 (32.1)	141 (33.4)	91 (33.0)	41 (40.2)*	30 (30.6)	8 (33.3)
rs3804100	T/C	97 (25.1)	155 (30.2)	134 (31.8)	84 (30.4)	39 (38.2)**	27 (27.6)	7 (29.2)
rs7656411	G/T	168 (43.5)	218 (42.4)	170 (40.3)	113 (40.9)	37 (36.3)	43 (43.9)	8 (33.3)

In the “Alleles” column, 1 indicates the major allele and 2 indicates the minor allele. *p=0.021, Pc>0.05 **p=0.013, Pc>0.06 These values are not significant after Bonferroni correction.

## Discussion

The current study was designed to determine whether *TLR2* polymorphisms affect the development of sarcoidosis in the Japanese population. Our results showed that all the *TLR2* polymorphisms so far examined were not significantly associated with any clinical subtype of sarcoidosis including ocular involvement in the Japanese population. However, a marginally significant p-value was observed for the SNPs rs3804099 and rs3804100 in patients with cutaneous manifestations, in comparison with the healthy control group. Healthy normal human skin contains two distinct major subsets of resident dendritic cells: Langerhans cells (LCs) and dermal DCs (DDCs). These DCs are located in the outer skin layers of the epidermis and play a critical role as the first line of defense against pathogens invading the skin. These DCs in the skin, especially DDCs, express TLR2 as well as TLR1 and TLR6. DDCs recognize bacteria and trigger the innate immune response [33]. Our results indicate that a possible connection may exist between *TLR2* polymorphisms and skin manifestations of sarcoidosis. The variants of the *TLR2* gene in DDCs may play a causative role in the development of cutaneous sarcoidosis in a site-specific manner.

Several reports have suggested that genetic variants of innate immune receptors might be associated with the risk of developing sarcoidosis [34]. Innate immune dysfunction caused by genetic factors may fail to eliminate pathogens. Consequently, it is postulated that frequent stimulation could lead to the chronic inflammation of sarcoidosis.

TLR4 is a major receptor for lipopolysaccharide (LPS), a component of gram-negative bacterial cell walls. TLR4 and TLR2 signal transduction results in the activation of inflammatory pathways involving nuclear factor-kappa B (NF-kB). Conflicting reports about the association between TLR4 and sarcoidosis have recently been published. It was shown that there is a significant association between patients with chronic sarcoidosis and *TLR4* polymorphisms in the Caucasian population [35]. Whereas, subsequent investigations by other groups found no significant association between polymorphisms and increased susceptibility to sarcoidosis [36,37]. Our group also could not find any association between *TLR4* polymorphisms and sarcoidosis in the Japanese population [29].

Nucleotide-binding oligomerization domain 2 (NOD2), a member of the NLR (Nod-like receptor) family, is an intracellular microbial sensor. This protein detects muramyl dipeptide (MDP); a component of bacterial peptidoglycans, and induces innate immune responses. Several reports have suggested there may be an association of polymorphisms in *NOD2* with early-onset sarcoidosis and Blau syndrome. However, no significant associations between the genetic polymorphisms in the *NOD2* gene and the risk of adult sarcoidosis were detected [36,38-41].

In the present study, genetic variations in TLR2 did not affect ocular sarcoidosis risk. However, some types of genetic predispositions underlying the pathogenesis of sarcoidosis can lead to ocular inflammation. Microbial pathogens have long been suspected as the cause of sarcoidosis. Therefore, further studies are needed to analyze other genes involved in the innate immune response against bacterial antigens.

In summary, the minor allele frequencies of *TLR2* do not appear to be significantly relevant to sarcoidosis in the Japanese population. However, in cutaneous sarcoidosis, rs3804099 and rs3804100 SNPs in *TLR2* are slightly associated with clinical disease. Further studies, especially in other ethnic populations, are required to elucidate what association there may be between sarcoidosis and *TLR2*.
